# Oncocytic carcinoma of the parotid gland with late cervical lymph node metastases: a case report

**DOI:** 10.1186/1752-1947-5-11

**Published:** 2011-01-17

**Authors:** Lorena Gallego, Luis García-Consuegra, Eduardo Fuente, Nicolás Calvo, Luis Junquera

**Affiliations:** 1 Department of Oral and Maxillofacial Surgery, Cabueñes Hospital, Gijón, Spain; 2 Department of Pathology, Cabueñes Hospital, Gijón, Spain; 3 University of Oviedo Dental School, Spain; 4 Department of Oral and Maxillofacial Surgery, Central University Hospital, Oviedo, Spain

## Abstract

**Introduction:**

Oncocytic carcinoma is a rare proliferation of cytomorphologically malignant oncocytes mainly found in glandular tissue, accounting for 0.5% of all epithelial salivary gland malignancies and 0.18% of all epithelial salivary gland tumors.

**Case presentation:**

We report a case of oncocytic carcinoma arising in the parotid gland of a 65-year-old Caucasian man. Our patient initially underwent left superficial parotidectomy, including the removal of the mass. A close follow-up was made, and four years after first surgery cervical lymph node metastases were confirmed. Therefore, a complete parotidectomy and radical neck dissections were performed. There were no complications and no sign of recurrence after six months of follow-up.

**Conclusion:**

Oncocytic carcinoma is an extremely rare malignancy in the salivary glands. Prophylactic neck dissection may be indicated for tumors larger than 2 cm in diameter (our patient's tumor was 2.5 cm at its greatest diameter). The clinical course of our patient, with the appearance of cervical lymph node metastases after four years of follow-up, supports this approach. Further investigation of the prognosis and correct treatment of patients with oncocytic carcinoma are required as more cases are reported.

## Introduction

Oncocytic carcinoma (OC) is an unusual proliferation of cytomorphologically malignant oncocytes and adenocarcinomatous architecture phenotypes mainly found in glandular tissue [1]. The terms oncocytic carcinoma, oncocytic adenocarcinoma, malignant oncocytoma and malignant oxyphilic adenoma are synonymous. Its malignant nature is distinguished from oncocytoma by abnormal morphological features and infiltrative growth [2]. Necrosis, peri-neural spread, pleomorphism, intra-vascular invasion, and distant metastasis to the cervical lymph nodes, kidneys, lungs, and mediastinum are the main features of this high-grade malignant tumor [1].

This tumor represents 5% of all oncocytic salivary gland neoplasms and less than 1% of all salivary gland tumors [3]. OC may occur in many sites in addition to the salivary glands, including the nasal and thoracic cavities, ovary, kidney, thyroid gland, breast and parathyroid. The majority of OC cases have occurred in the parotid glands, but tumors that involved the submandibular gland and minor glands of the palate have also been described [4].

In this case report we describe a case of OC that arose in our patient's parotid gland and presented regional lymph node metastases four years after first surgery.

## Case presentation

**Figure 1:**
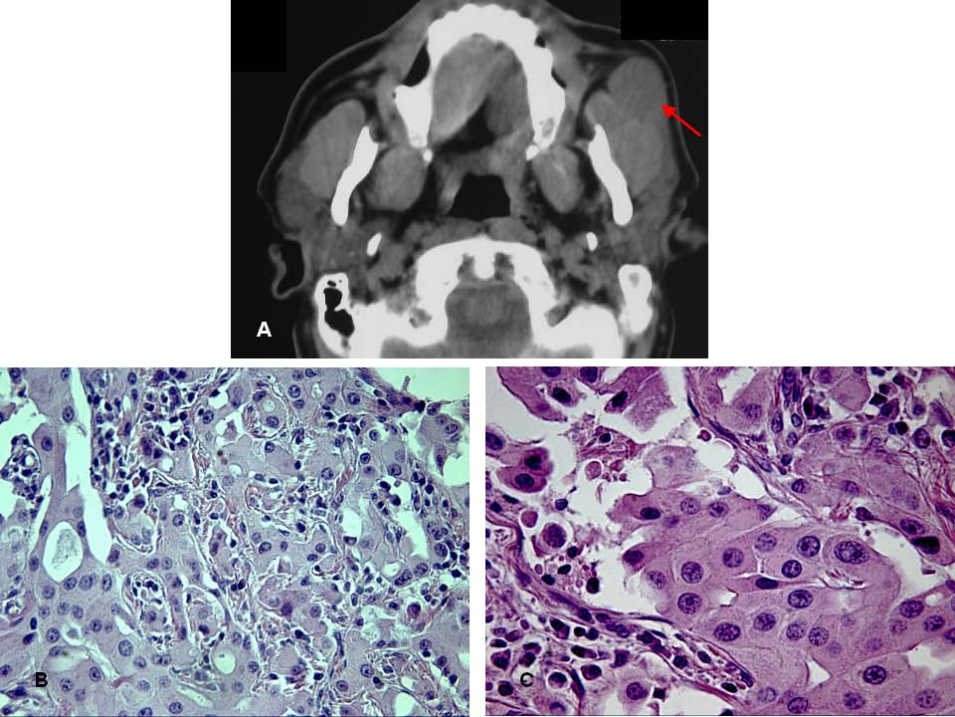
**A) Computed tomography image revealing a well demarcated mass in the anterior portion of the left parotid gland resembling a mixed tumor (red arrow)**. B) Histopathological image showing peri-neural invasion by the tumor and cohesive clusters of neoplastic cells (hematoxylin and eosin stain, original magnification 250×). C) High magnification image demonstrating oncocytic cells with abundant and finely granular cytoplasm and moderately pleomorphic nuclei located centrally or peripherally (hematoxylin and eosin stain, original magnification 400×).

A 65-year-old Caucasian man presented with a one-year history of a painless mass in the left parotid gland. Physical examination revealed an elastic, movable and non-tender mass. There was no facial palsy or regional lymphadenopathy. A computed tomography (CT) scan revealed a 20×25 mm mass in the anterior portion of the gland, near the parotid duct, resembling a mixed tumor (Figure 1A). The cervical and peri-aortic lymph nodes were not enlarged. No calcification or cystic component was seen within the tumor. Fine-needle aspiration cytology (FNAC) results showed no signs of malignancy.

A month later, our patient underwent left superficial parotidectomy, including the removal of the mass. Because of clear infiltration of the tumor, the buccal branch of the facial nerve was resected. Macroscopically, the tumor was a well-circumscribed, firm, grey-brown, ovoid nodule measuring 2.5 cm in diameter. Microscopic examination revealed that the neoplasm had replaced a wide area of the parotid gland and peri-neural invasion was found (Figure 1B). The neoplastic elements were large, round or polyhedral cells and were arranged in solid sheets, islands and cords. The cytoplasm was abundant, eosinophilic and finely granular, characteristics indicative of oncocytes (Figure 1C). A diagnosis of OC was based on these histopathological findings and in particular on the invasive growth pattern.

**Figure 2:**
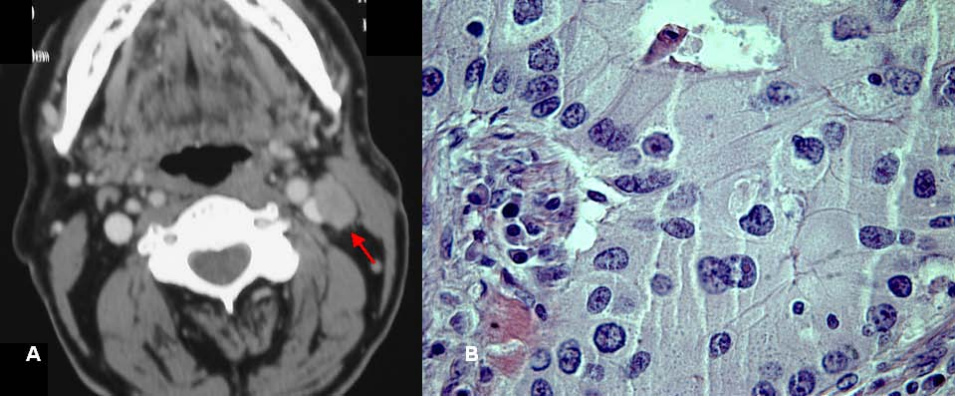
**A) Computed tomography image four years after first surgery revealing a cervical lymph node enlargement (red arrow)**. B) Histopathological study of cervical specimen showing metastatic tumor infiltration by oncocytic cells (hematoxylin and eosin stain, original magnification 400×).

We preferred a close follow-up to an elective neck dissection, and reserved the neck dissection for a recurrence, as there was no lymph node involvement either clinically or radiologically. Our patient was followed up accordingly, and four years after surgery started complaining of swelling and pain in the left cervical area. Physical examination revealed an elastically hard and poorly defined left cervical mass. There were no signs of local recurrence. FNAC showed oncocytes and atypical cells. A CT scan demonstrated several enlarged lymph nodes in the left parotid gland and on the left side of the neck (Figure 2A). Therefore, a complete parotidectomy and radical neck dissection were performed. A microscopic examination of tissue samples confirmed that three lymph nodes were positive for metastases, as they had the same histopathological features as the primary tumor (Figure 2B). Post-operatively there were no complications and no sign of recurrence after six months follow-up.

## Discussion

OC is an extremely rare malignancy in salivary glands, accounting for just 0.5% of all epithelial salivary gland malignancies and 0.18% of all epithelial salivary gland tumors [5]. Criteria for the diagnosis of malignancy in salivary oncocytic tumors include: (1) local lymph node metastasis; (2) distant metastasis; (3) peri-neural, vascular or lymphatic invasion; (4) frequent mitoses and cellular pleomorphism with extensive invasion and destruction of adjacent structures [6]. This tumor is predominantly composed of round or polyhedral cells arranged in small clusters and occasional solid sheets. Cells have abundant eosinophilic cytoplasm, a result of excessive numbers of mitochondria [1]. Although not commonly used, it has been reported that anti-mitochondrial antibody is highly specific and sensitive to confirm the oncocytic nature of the granular cytoplasm [7]. FNAC is less sensitive for oncocytic neoplasms, perhaps due to the rarity of these tumors and diagnostic pitfalls previously associated with FNAC (for example, sampling errors and overinterpretation of paucicellular specimens) [2].

Approximately one-third of patients with oncocytic carcinoma of the parotid develop a painful mass or experience facial paralysis. The skin overlying the gland is occasionally discolored or wrinkled [1]. In our case, there was no facial paralysis or discoloration of the overlying skin. The average age of patients has been estimated to be about 60 years, with a male predominance [1,8].

As with other high-grade salivary gland neoplasms, aggressive surgical intervention is indicated. Therapy for OCs consists of surgical intervention, and total parotidectomy with preservation of the facial nerve whenever possible. The efficacy of radiotherapy is unclear. Radiation does not appear to favorably alter the biological behavior of this tumor. In the case reported, intra-operative frozen sections of the tumor would have been indicated for pathological diagnosis and would have altered the surgical decision. FNAC alone did not determine a correct diagnosis and the first surgical management was inadequate.

A number of studies have reported multiple recurrences of this tumor and regional or distant metastases [2,6,8-10]. The cervical lymph nodes may be affected as with other malignant tumors of the parotid gland, but prophylactic neck dissection must be considered individually in the absence of consensus in the literature. Nakada *et al*. published a review of 28 cases of oncocytic carcinoma of the parotid gland [8]. They concluded that distant metastasis appeared to be the most important prognostic feature of oncocytic carcinoma; local lymph node metastasis was not necessarily a critical factor in the overall prognosis. Distant metastasis sites include the lung, liver, kidney, mediastinum, thyroid gland and bone.

Prophylactic neck dissection may be indicated for tumors larger than 2 cm in diameter [10] (our patient's tumor was 2.5 cm at its greatest diameter). This likely indicates a worse prognosis. The clinical course of our case, with the appearance of cervical lymph node metastases after four years of follow-up, supports the approach of Goode and Corio [10].

The prognosis of oncocytic carcinomas is not well known because of their low incidence. Patients with malignant oncocytoma appear to have good short-term survival, but poor long-term survival [11]. The average survival period has been estimated at 3.8 years with metastasizing tumors [12]. Further investigation of the prognosis and correct treatment of patients with OC are required as more cases are reported.

## Conclusion

OC is an extremely rare malignancy in salivary glands, and standard treatment and prognosis is unclear. Clinicians should perform a careful follow-up, as distant metastasis appeared to be the most important prognostic feature.

## Consent

Written informed consent was obtained from the patient for publication of this case report and any accompanying images. A copy of the written consent is available for review by the Editor-in-Chief of this journal.

## Competing interests

The authors declare that they have no competing interests.

## Authors' contributions

LG was a major contributor to the writing the manuscript. LG-C was the main surgeon, reviewed our patient's notes, collected the data and prepared the radiology slides. ED performed the histological diagnosis and photographs. NC and LJ reviewed and corrected the manuscript. All authors read and approved the final manuscript.

## References

[B1] Ellis GL Auclair PL Tumors of the salivary glands Atlas of Tumor Pathology 2008 4 Washington, DC: Armed Forces Institute of Pathology 356 363 fascicle 9

[B2] Capone RB Ha PK Westra WH Pilkington TM Sciubba JJ Koch WM Cummings CW Oncocytic neoplasms of the parotid gland: a 16-year institutional review Otolaryngol Head Neck Surg 2002 126 657 662 10.1067/mhn.2002.124437 12087334

[B3] Barnes L Eveson JW Reichart P Sidransky D Pathology and genetics of head and neck tumors World Health Organization Classification of Tumors 2005 Lyon, France: WHO 235

[B4] Guclu E Oghan F Ozturk O Alper M Egeli E A rare malignancy of the parotid gland: oncocytic carcinoma Eur Arch Otorhinolaryngol 2005 262 567 569 10.1007/s00405-004-0871-4 15592856

[B5] Giordano G Gabrielli M Gnetti L Ferri T Oncocytic carcinoma of parotid gland: a case report with clinical, immunohistochemical and ultrastructural features World J Surg Oncol 2006 4 54 10.1186/1477-7819-4-54 16923179 PMC1564019

[B6] Gray SR Cornog JL Jr Seo IS Oncocytic neoplasms of salivary glands: a report of fifteen cases including two malignant oncocytomas Cancer 1976 38 1306 1317 10.1002/1097-0142(197609)38:3<1306::AID-CNCR2820380333>3.0.CO;2-A 953970

[B7] Shintaku M Honda T Identification of oncocytic lesions of salivary glands by anti-mitochondrial immunohistochemistry Histopathology 1997 31 408 411 10.1046/j.1365-2559.1997.2870891.x 9416480

[B8] Nakada M Nishizaki K Akagi H Masuda Y Yoshino T Oncocytic carcinoma of the submandibular gland: a case report and literature review J Oral Pathol Med 1998 27 225 228 10.1111/j.1600-0714.1998.tb01946.x 9682986

[B9] Cinar U Vural C Basak T Turgut S Oncocytic carcinoma of the parotid gland: report of a new case Ear Nose Throat J 2003 82 699 701 14569705

[B10] Goode RK Corio RL Oncocytic adenocarcinoma of salivary glands Oral Surg Oral Med Oral Pathol 1988 65 61 66 10.1016/0030-4220(88)90193-4 3422397

[B11] Ardekian L Manor R Peled M Laufer D Oncocytic neoplasms of the parotid gland: a 16-year institutional review J Oral Maxillofac Surg 1999 57 325 328 10.1016/S0278-2391(99)90682-1 10077205

[B12] Brandwein MS Huvos AG Oncocytic tumors of major salivary glands. A study of 68 cases with follow-up of 44 patients Am J Surg Pathol 1991 15 514 528 10.1097/00000478-199106000-00002 2031528

